# "The Hospital" Nursing Mirror

**Published:** 1897-08-21

**Authors:** 


"The Hospital" Nursing Mirror.

Being the Nursing Section of "The Hospital."

[Contributions for this Section of "The Hospital" should be addressed to the Editor, The Hospital, 28 & 29, Southampton Street, Strand, London, W.C., and should have the word "Nursing" plainly written in left-hand top corner of the envelope.]

### News from the Nursing World.

### A SUCCESSFUL TRAINING SCHOOL FOR NURSES.

The Prince Alfred Hospital, Sydney, has trained a long list of successful matrons and nurses working in New South Wales. Miss Blanch Bricknell has recently been appointed matron of the Carrington Hospital for Convalescents at Camden. The salary attached to this post is £150 per annum, and it is regarded as one of the "plums" of the profession. Miss Bricknell was seven years at Prince Alfred Hospital, for four and a half years of which time she was sister. She was appointed matron of Deniliquin Hospital not very long ago, so her promotion has been rapid. She was selected out of 45 candidates from all parts—Victoria, Queensland, and Tasmania—to fill her present position. Another recent appointment is that of Miss Pope, one of the "Sisters" P. A. H., to be matron of Rydalmere Hospital for the Insane. The majority of important hospital posts in Sydney and the suburbs are filled by nurses trained at this hospital; for instance, Miss Gould, matron of the Sydney Hospital; Miss McMasters, matron of the Coast Hospital; Miss Ryrie, matron of "Craigend House," the principal private hospital; were nurses at Prince Alfred Hospital before the advent of the present able matron, Miss McGahey. The matrons of Manly Cottage Hospital, Dr. George Cottage Hospital, the Western Suburbs Cottage Hospital, and the Paramatta Hospital have also been trained at the P. A. H.

### MEDALS AND PRIZES FOR THE NURSES AT BRADFORD.

The following probationer nurses have distinguished themselves at the annual medical and surgical examination at Bradford Infirmary: Gold medal, Nurse Tanner 422 marks); silver medal, Nurse Jasper (356); Nurse Fawcett (344), Nurse Bailey (328), Nurse Pearson (325), Nurse Oliphant (313), Nurse Butterworth (312), Nurse Brough (290), Nurse Raspin (274), and Nurse Turpin (271). The highest number of marks possible to obtain was 450. The Mayor and a large number of ladies and gentlemen interested in the infirmary assembled in the board-room on the occasion of the medals and prizes being awarded, and several speeches of a complimentary character were made.

### A BRAVE NURSE.

The courage of woman is, as a rule, a passive quality. A nurse must constantly face death in pursuit of her calling, and does so with an almost perfect indifference to the danger incurred; but it is otherwise when the peril comes in a sudden, noisy, and confusing way, therefore Nurse Beadon's conduct during the late earthquakes in India is the more admirable. When the first shock was felt she was in attendance on a delirious patient, a tall, muscular man, who immediately sprang out of bed and rushed to the door. The nurse had barely time to drag him back to bed before the ceiling fell. She managed to protect him from the falling bricks by her own body, which was badly hurt. Then, the shock over, and finding the door blocked, she dragged him to the window, and pushed him into the arms of an attendant standing below. Although the house was still rocking in a most alarming way, she secured all the bedding and clothes before quitting it. Neither nurse nor patient fortunately were much the worse, the result of a woman's presence of mind and pluck. The charge of a delirious, muscular man, however, requires equal courage, but people don't think of that, consequently there are no facilities offered to men who wish to train for such work.

### THE BOROUGH NURSES' HOME, PAISLEY.

The new home which is being built for the nursing staff maintained by the trustees of the late Mr. Peter Borough is nearing completion, and will be opened by the Marchioness of Lorne when she visits Paisley in October. The site is a good one, being on sloping ground opposite the Coats Observatory, and commanding an extensive view. The architecture is solid and homely, and the building, of red stone, is two stories high. The top floor is arranged for the accommodation of three nurses, and the domestics with one room set apart for sickness. On the floor beneath are six bedrooms; on the ground floor are the entrance hall and office, drawing-room, dining-room, and matron's sitting-room. The kitchens, store and cloak rooms are relegated to the basement. Each floor is provided with a bath-room. The partition between the drawing and dining rooms is removable, so that the two rooms can be converted into one when occasion requires it. The building is wired for electric light, and care has been given to the ventilation. Large and well laid-out recreation grounds are situated in the rear of the building, which are accessible from the drawing-room balcony. The architect is Mr. T. Graham, Abercrombie.

### CO-OPERATION "AT HOME."

Miss Philippa Hicks was presented by the members of the Nurses' Co-operation with a silver tea service, and by the staff with a complete set of table silver, in recognition of her six and a half years' work as first lady superintendent, at a successful and enjoyable "At Home" given in the Medical Society's rooms, 11, Chandos Street. A good programme of instrumental and vocal music was given by Miss Kate Kimbell, A.R.C.M., Miss Arabella Wood, Mr. W. Beauford Mitchell, Miss Edith Ravenhill, and Miss Hetty Kimbell. Miss Gethan, the secretary, told a story, entitled "Across a Cot," with great spirit. A large number of nurses were present.

### A CHINESE LADY DOCTOR.

The fascination exercised by the profession of medicine over women has reached that country noted as possessing the oldest civilisation, yet as being mos behind the times. The granddaughter of a wealthy Chinese mandarin, Miss Hu King Eng, has taken an

M.D. diploma in America, and is now in charge of the Siang-Hu Hospital at Foo-Chow. This lady has performed some most successful operations, and if the Women's Congress be held in London next year she will probably be one of the delegates. Both she and her grandfather are Christians.

### THE NURSES' HOLIDAY.

In honour of the Queen's Record Reign the matron, nurses, and maids of Cardiff Infirmary have been granted an extension of holiday leave. It appears from information recently received that before the question of longer leave was mooted the senior nurses applied to the matron, in the first instance, for a longer holiday, and, as she did not see her way to comply with their request, they carried it before the committee, with the result that, after special inquiry had been made, it was granted, and this without provoking the slightest ill-feeling in any quarter.

### SICK NURSING AT MORPETH.

The first sick nursing class for women at Morpeth has just reached a successful close. All of the twenty members who presented themselves for examination passed, and Mrs. M. F. Atkinson and Miss U. S. Angus both took silver medallions. Dr. Hugh Dickie, M.A., was lecturer and demonstrator, being helped in the latter work by Nurse Morton. The class was instituted, under the St. John Ambulance Association, by the local Technical Education Committee.

### AN APPEAL FOR ANGLESEY.

Anglesey is to have a Victoria Nursing Association to commemorate the year, if Mr. R. Williams Bulkeley, H.M.'s Lieutenant for the County, can raise the money. For this purpose he makes a strong appeal through the press. The idea is to establish a central association, which should supply nurses free to the needy, lend help to local associations in times of stress, and supply private nurses when required on suitable terms. The money collected is to be invested as capital, and the interest used to support the nurses.

### THE BIRKENHEAD JUBILEE MEMORIAL.

The goal set before the inhabitants of Birkenhead as a suitable memorial of the Queen's long and glorious reign was the addition of two new wards to the Borough Hospital, and the erection of a nurses' home at the estimated cost of £6,000. The result has been to show how very small a portion of the community bear the brunt of supporting the far-reaching and universally beneficent medical charities, and that that portion belongs to a class which needs them least. At Birkenhead the sum of £4,300, which has been subscribed of the sum asked for, has been the gift of 261 persons. It is instructive to remember that this is a flourishing town, which has a dispensary with a roll call of 4,560 patients, one hospital with 60 beds, a woman's hospital with 20 beds, and a children's hospital with 36 beds, all of which implies a large population dependant on the hospitals for medical attendance, and a considerable number of prosperous inhabitants well able to take care of themselves, and who might not unreasonably be supposed would be glad of the opportunity to help their poorer neighbours in such a practical and useful way. As matters stand now the nurses' home is to be built at a cost of £1,500, and one ward only is to be added to the hospital, and it is to be hoped that before the fund closes at the end of the autumn the £800 necessary to complete even this narrowed scheme will be freely subscribed.

### JUBILEE REJOICINGS IN SYDNEY HOSPITALS.

Prince Alfred Hospital, which was built to commemorate the recovery of the Duke of Edinburgh after his attempted assassination, was gaily but inexpensively decorated with bunting and garlands; and Miss Thompson and Miss Harris, two well-known ladies, gave the patients a light extra tea. The patients at the Carrington Hospital also enjoyed an extra tea, with an evening concert afterwards. The illuminations were effective and much admired, and, as this hospital is situated on a hill, they were seen from the town of Camden, two miles away.

### REPORT OF THE COREAN MISSION.

The sixth report of the work connected with Bishop Corfe's mission is issued. It is a record of success throughout, bought, as all success must be, at the cost of trouble and anxiety. The first gain is the growing appreciation with which the Coreans regard the services of the doctors and sisters at the hospitals and dispensaries of Chemulpó and Seoul, and their eagerness to receive such help in sickness. The hospital accommodation is, indeed, too limited, and some four thousand patients were treated during the nine months ending September, 1896. St. Peter's Hospital for Women and Children is in charge of a lady doctor and five sisters, and is the centre of all kinds of philanthropic work apart from that of a hospital. The sisters are engaged in caring for orphans, succouring the aged and destitute, and teaching the many who are willing to be taught. Some villages petition for schools and schoolmasters, promisisg to send their children, and others offer their children as presents on the condition that they are educated! On every side the work is extending. The need of trained nurses is felt, but a want of adequate funds prevents the mission from supplying them at present. The little girls enjoy being taught to sew, and acquire the art quickly.

### SHORT ITEMS.

At Stoke Damerel Workhouse the nurses are to be made to understand clearly that they are responsible—(1) To the doctor for the medical treatment of the patients in the infirmary; (2) to the master and matron in other matters; (3) under the regulations of the Local Government Board; and the Guardians are happily confident that "there will be no friction or unpleasantness in future." What! with a threefold and possibly antagonistic authority?—Miss Mabel Thomas makes an appeal to lady cyclists for their discarded machines. There are five nurses working in Pevensey and district, the cottages are poor and widely scattered, and the association a poor one. Under such circumstances the great use of bicycles to the nurses and neighbourhood is obvious.—The new Victoria Nursing Institute, Wellington, has for trustees Messrs. J. H. Fox, O. G. Walter, and E Corner. The General Committee was also elected on August 6th at a meeting of donors and subscribers held in the Town Hall.—A committee has been appointed to arrange a course of sick nursing for the pupils of the Board school at Heckmondwike.—The Guardians are so divided upon the subject of spending £3,500 upon the nursing home at Prescot, that the scheme was only carried by the reluctant vote of the chairman. There are eight nurses now on the staff, and the new home will provide accommodation for twenty-seven.

### Practical Aspects of a Nurse's Life.

By Sister Grace.

### V.—BEDSORES—THEIR PREVENTION AND CURE.

In former days bedsores were considered a natural and expected addition to the trials of a long illness; now, we consider it more or less a disgrace to the nurse if her patient develops such a thing, except in exceptional cases. All nurses have their own favourite theory and practice as to the prevention and cure of these dreaded visitors. Most ward sisters keep a watchful eye on the backs of all their patients themselves, but they are naturally obliged to leave much of the actual treatment to others. I made a rule that the backs of all patients confined to bed entirely should be attended to three times a day, and would not allow this to be done (except in cases of extreme difficulty) when the beds were made morning and evening, because when the pressure of work is great there is a natural tendency to hurry over this most necessary duty if it is done when nurses are anxious to get beds made and the ward tidy. Perfect cleanliness is one of the greatest safeguards—in cases where the patient is helpless from a fractured thigh or other injury, or in cases of dysentery, typhoid fevor, &c., when bed-pans are necessarily in frequent use, the greatest care is required. Be conscientious in carrying out all orders you receive on the subject; if the staff nurse tells you that a case requires attention to his back every four hours, she does not mean that you should put a little ointment on your hand and smear it on the back and hips, but that you should rub steadily for at least two minutes, wash the back if necessary, pull the drawsheet through, and see that there is no crease or crumb under the patient.

When the sufferers are in a chronically wet condition the difficulties are greatly increased, but it is imperative that they should be kept as dry as circumstances will permit; therefore do not wait to be told that a bed wants attention. You will find as your experience widens that different skins require different treatment. Some which are naturally rather dry, as in the case of the aged, do not respond well to the use of spirit; others do not answer well with ointments, as there is a tendency to develop eczema, and will do well if steadily rubbed only with soap on the hand. Remember that the object you have in view is to restore circulation, which is impeded by continual pressure on the bed. To this end friction with a steady and slow circular movement of the hand will do more to prevent bedsores than all the ointments in the world. The chief use of any unguent is to make the friction easier and pleasanter. Hospital patients themselves have great faith in what they call "grease," and think that if a little is good much must be better, in pursuance of which theory I found an old man picking the butter out of his bread at tea with a hedger's knife. On my inquiring what he was doing, "No harm, sister; I am just hucking out the grease (pronounced 'grace') to rub on my back; I finds it comforting like when you puts it on." If in spite of all your care a dark spot appears, lose no time in showing it to someone in authority.

Sometimes patients are brought into the hospital with truly appalling bedsores, indeed of a magnitude hardly to be believed without occular proof, and very difficult they are to cure when once they have taken possession. In cases like these, which have come from neglect and lack of treatment altogether, the first thing necessary is to get them clean and healthy by the use of fomentations, then if there is a slough which baffles your efforts to dislodge it, I have found them yield to an application of Peruvian balsam. When the slough has come away, the balsam must be discontinued, as it is too stimulating. Plain castor oil is as good a remedy as any, and after a time it is a good plan to change it to red lotion. My own idea is that rather frequent change of treatment is beneficial, not wedding oneself too obstinately to one favourite method.

Whether bedsores are more to be feared in medical or surgical work is a vexed question. Speaking broadly, I think that medical nurses have the greater difficulties to contend with, because their patients are in a more or less bad state of health, whereas surgical patients, except such cases as hip and spine disease, and some forms of malignant disease, are generally fairly robust. In cases of dropsy and paralysis, for instance, the difficulties are very great; in the former the patients are often extremely heavy and difficult to move, whilst the condition of the skin is sadly favourable to the development of bedsores, and in the latter everything adds to the original danger. In typhoid fever, again, the patient often becomes extremely emaciated, and the perspiration from its acrid character has an irritating effect on the skin; added to this, in many cases the evacuations are passed in the bed.

There are other difficulties which beset the surgical nurse, some of them very serious ones, such as battling with a curious form of sores which develop very rapidly in surgical cases of old standing, when the blood has become vitiated; these for lack of another term are sometimes called bedsores, because they appear wherever the body rests on the bed, but they come in other localities as well. In the present day splints are so ingeniously contrived that the difficulties of moving patients suffering from fractures, hip disease, &c., are greatly reduced, but on the other hand some surgical cases it is important to keep so absolutely immovable that the ingenuity of the nurse is greatly taxed to preserve the back in a faultless condition.

No one can overestimate the importance of the greatest attention to this detail of nursing; it is to my mind one of the distinguishing marks of a thoroughly first-class nurse.

### Minor Appointments.

The Infirmary, Rochdale.—Miss E. Brandon and Miss M. D. Bryan have been appointed Charge Nurses in this infirmary. Miss Brandon was trained in the Royal South Hants Infirmary, Southampton, and has been staff nurse in the same institution, and also in the New Hospital for Women, London. Miss Bryan was trained in Sir Patrick Dunn's Hospital, Dublin, and has been staff nurse at the City Hospital, Liverpool, and at Erdington, and sister of the City Hospital, Birmingham.

Park Hospital, Hither Green, Lewisham. — Miss Henrietta Tarleton Young and Miss Lucie M. Todd have been appointed Charge Nurses at the new Park Hospital, Hither Green, Lewisham. Both ladies were trained at Addenbrooke's Hospital, Cambridge, Charing Cross Hospital, and the City of London Lying-In Hospital, City Road, and hold the L.O.S. diploma.

Homerton Fever Hospital.—Miss Hinda Herbert has been appointed Charge Nurse at the Homerton Fever Hospital. Miss Herbert received a three years' training at the Cumberland General Infirmary, and for the last year has held the post of ward nurse at the Infirmary, Isleworth, W.

Royal Infirmary, Windsor.—Miss M. E. Yelland was appointed Night Sister of this infirmary on August 5th. She was trained at the Dewsbury General Infirmary, and had previously been staff nurse at the Ear and Throat Hospital, Shrewsbury, and also staff nurse at the Metropolitan Hospital, Kingsland Road, N.E.

### The Nursing of the Sick in Workhouses.

THE long expected General Order as to the nursing of the sick in workhouses has at last been issued by the Local Government Board. It is a most satisfactory document in many respects. No doubt it has its weak points. The superintendent nurses will still have to act under the direction of the master or matron in all matters except those concerned in the treatment of the sick, and a great deal of the utility of the Order will depend upon the interpretation officially given to the phrase "in all matters of treatment of the sick." Again, in the numerous small workhouses in which there are only one or two nurses, although they will in future have to be trained, the hardship of their present relation to the master or matron does not seem to be at all alleviated. Still in many ways the Order is a tremendous advance on the present state of affairs, and the institution of superintendent nurses is an especial source of gratification to those interested in workhouse nursing.

Article I. lays down that "no pauper inmate of the workhouse shall be employed to perform the duties of a nurse in the sick or lying-in wards of the workhouse, or be otherwise employed in nursing any pauper in the workhouse who requires nursing." It also states that "no pauper inmate of the workhouse shall be employed as an attendant in the sick or lying-in wards of the workhouse, or upon any pauper in the workhouse who requires nursing, unless such inmate shall be approved by the medical officer of the workhouse for the purpose, and shall act under the immediate supervision of a paid officer of the Guardians."

At first sight these provisions seem satisfactory, but a good deal will depend upon interpretation, especially in the latter clause. The difference between "nursing" and being "employed as an attendant" upon a "pauper . . . who requires nursing," is somewhat obscure, and it is to be noted that the "immediate supervision" under which this attendant acts is not necessarily that of a nurse but of a "paid officer"—maybe the matron.

Article II. deals with training, and provides that no person shall be appointed "to the office of nurse or assistant nurse" without having "had such practical experience in nursing as may render him or her a fit and proper person to hold such office." Here, again, much will depend on what the Local Government Board shall define to be "such practical experience," &c. It is provided that this article shall not apply to the case of a "female assistant nurse" where there is a superintendent nurse. The object of this exception is to allow of the employment of probationers, but it is to be noted that the effect of the article is to abolish probationers except where there is a superintendent nurse; it also makes it impossible for male nurses to be trained in workhouses.

Article III. lays down that where the staff of female nurses and assistant nurses consists of three or more persons the Guardians shall either appoint a superintendent nurse "or, with our consent, direct that one of the nurses shall be a superintendent nurse." Where at the commencement of this Order, *i.e.*, September 29th, 1897, there shall not be a staff of three female nurses and assistant nurses, and it is proposed that there should be such a staff, and also where any superintendent nurse ceases to hold office, the Guardians shall appoint a superintendent nurse. Finally, any superintendent nurse appointed after the commencement of this Order, *i.e.*, September 29th, "shall, unless we dispense with the requirement, be a person qualified for the appointment by having undergone for three years at least, a course of instruction in the medical and surgical wards of any hospital or infirmary being a training school for nurses, and maintaining a resident physician or house surgeon."

According to this article, then, the first appointment on the commencement of the Order may be made from the existing staff, irrespective of the length of training, but after that date every superintendent nurse must have had at least three years "instruction." It should be noted by some of our training schools that "instruction" for one year and experience alone for the remaining two years will not qualify for the post of superintendent nurse. Nurses undergoing training will do well to make special note of this.

Article IV. gives to the superintendent nurse the control of the other nurses and assistant nurses, "subject to the directions of the medical officer of the workhouse," in all matters of treatment of the sick, but "in all other matters to the directions of the master or matron of the workhouse, so far as the orders in force in the Poor Law Union and the lawful directions of the Guardians may require or permit." A very important clause is the following: "No such superintendent nurse shall be dismissed without our consent."

Article V. provides that if it appears to the medical officer that a temporary nurse is required in an emergency and he informs the master of the workhouse in writing accordingly, it shall be the duty of the master to engage a person to act as nurse until the next meeting of the Guardians, who shall pay the reasonable remuneration of the nurse so engaged. Where there is no superintendent nurse no person shall be engaged under this Article without having had such practical experience in nursing as may render him or her a fit and proper person to hold the office of nurse. All temporary help will then have to be of the same standard of efficiency as the regular nursing staff.

Article VI. provides that this Order shall not apply to any infirmary or school which is under administration separate from the workhouse.

We need hardly say how great an advance in the nursing of the sick in workhouses is likely to result from the application of this order, if only a sufficiently high standard is given to the definition of the terms employed. The Order may be cited as "The Nursing in Workhouses Order, 1897."

### The King of Siam and the Children.

THE King of Siam is a great lover of children, and has shown his interest in them by visiting the Child's Hospital, Edinburgh. He went to nearly every little cot, and asked the little occupants how they were, or if they suffered pain, and bade them be quick and get well. Hearing that the convalescent children in the nursery would be disappointed at not seeing him, His Royal Highness, in spite of having already stayed longer than he intended, mounted to the top of the building to "let the children see the King." The object of the visit may have been something more than merely sight-seeing, for the King has established hospitals in his own land for the suffering little ones.

### Appointments.

### MATRONS.

NORTH RIDING INFIRMARY, MIDDLESBROUGH.—On August 1st Miss Emily S. Nowers was appointed Matron of this institution. She was trained at the London Hospital, and has been lady superintendent of the in-patient department, Birmingham and Midland Hospital for Women, Sparkhill, Birmingham.

### Presentation to Miss Philippa Hicks.

The Nurses' Co-operation gave an "At Home" on Tuesday, August 17th, at the Medical Society's Rooms, 11, Chandos Street, Cavendish Square, on the occasion of presenting Miss Philippa Hicks, the first lady superintendent, with a silver tea service and a complete set of table silver. Over a hundred nurses were present, and refreshments and music were provided.

After the formal business was concluded, the Chairman (J. P. Bickersteth, Esq.) made a speech. He expressed the universal regret felt by the management and staff that Miss Hicks' state of health compelled her to resign her position as lady superintendent of the Co-operation. Miss Hicks was fully acquainted with all matters appertaining to nursing. She was trained at the Nightingale School for Nurses, and thence went to the St. Marylebone Infirmary, and then to the Brompton Consumption Hospital. In 1885 she took charge of the army nursing in the Egyptian campaign, working with the Princess of Wales's nurses. On her return to England she took up work as home sister, and later as assistant matron at King's College Hospital, and then she became lady superintendent of the Children's Hospital, Great Ormond Street. In 1891 she was invited to become first superintendent of the Nurses' Co-operation, newly founded, and with a staff of thirty nurses. Next year the staff increased six-fold, and now there are between four hundred and five hundred names on the register, and there is room for more, for work is plentiful. The Chairman made the remark that the one defect in the Nurses' Co-operation was the want of a residential home, and that will possibly soon be remedied, Miss Hicks being in correspondence with a wealthy and benevolent lady with a view to supplying the need. Miss Hicks does not sever her connection with the society, she becomes a member, and it is intended to propose her name as vice-president at the next general meeting. He concluded by asking her to accept the gifts from the members of the association and from the staff expressing the pleasure he experienced in being deputed to do so.

Nurse Leigh proposed a vote of thanks to Miss Hicks, in which she said every nurse joined, and she also bore a message to Miss Hicks from the nurses to the effect that they hoped she would remember that the first Friday in every month was their "At Home" day, and that she would always be a welcome guest.

Sir Henry Burdett seconded this vote. He reminded those present that had it not been for Miss Hicks there would have been no co-operation at all. When Nurse Belcher (a lady since married) came forward to urge a scheme whereby the nurses should have their own earnings, it provoked strenuous opposition. This arose from a mistaken idea on the part of those who were responsible for the existing system. Those present could understand that the nurses owed the constituted authorities obedience and respect, and if they were to make their voices heard in spite of them, the captain of the new ship must have great courage. Many ladies refused the position because they were afraid, but Miss Hicks took the helm, and with the successful commencement of the Nurse's Co-operation closed the first chapter of the first chronicle of co-operative nursing. It is only right that the public should know, that the future historians of the movement should know, how much courage and self-denial were needed on the part of the lady who became first superintendent. He said he could illustrate this best by telling them of a matron whom he revered for her knowledge of nursing and the admirable way in which she fulfilled the duties of her position. This lady wrote to him in a letter, which did she read it now would provoke a smile, to the effect that if he persisted in this scheme "we will rule you out of all the training schools." The movement rose out of a keen, intense desire, as well as a keen and intense necessity for co-operation. There were 25,000 nurses in England alone. The risks attendant on the career of a private nurse are great, even with co-operation, and few nurses dare face them alone. The pecuniary advantage of private nrusing in an institution of private nurses is not much less than that offered by the Co-operation. On an average the private institution nurse earns £80 a year, whilst the co-operative nurse £100, a difference only disinfection, for rest, and for nursing and attention if the nurse contracts sickness from her patient. The Co-operation had been a surprising success, and the best way in which the staff could show Miss Hicks' appreciation of her services was by each individual nurse resolving never to do anything that would throw discredit on the society. In conclusion, Sir Henry pointed out that there was only one Nurses' Co-operation by Act of Parliament, *i.e.*, the Nurses' Co-operation, 6, New Cavendish Street, W.; but there were many attempts in many places to imitate it, attempts which were often nothing more than private venture houses run for the benefit of the proprietor, and the public ought to be warned to make inquiries of each of these societies and find out if it was governed by a committee, and, if so, who were upon that committee, before employing nurses through its agency.

Miss Hicks, in thanking the assembly, said she found it very difficult to speak of what she felt in receiving these gifts. She believed that these gifts were the outcome of real honest friendship. She had found that her six and a half years' work had been the means of her making many new friendships and cementing old ones. The public had seen the success of the Co-operation. She and her nurses had known the difficulties, but there is nothing like facing difficulties in combination for binding all together. Miss Hicks ended heartily with "Thank you all ever so much for all you have given me and all you have been to me."

A vote of thanks was given to the chairman, who has never been absent from one committee meeting. The proceedings were marked by great cordiality and were altogether successful.

### Hospital Stamps in New South Wales.

The Executive Committee of the "Proposed Victoria Homes for Consumptives Fund" is still very energetic, and subscriptions are coming in steadily. Several cheques were received from warehousemen, in lieu of illuminating. Lord Hampden's suggestion that a special stamp be issued has been agreed to by the Government, and 40,000 shilling stamps, to be used as 1d. postage, and 10,000 half-crown stamps, to be used as 2½d., are to be issued. The sale of these will considerably augment the Fund.

### A Nursing Home for Children.

A new home has recently been opened at Worth, near Three Bridges, Sussex, by Mrs. Henrietta Law (Sister Henrietta) for the nursing and care of delicate children. Rushmoor is situated about a mile from Three Bridges, in a healthy and beautiful neighbourhood, the house is roomy, the grounds large, and the appointments good. A liberal supply of country delicacies, milk, eggs, fruit, and vegetables, add greatly to its attractions from a medical point of view.

### Everybody's Opinion.

[Correspondence on all subjects is invited, but we cannot in any way be responsible for the opinions expressed by our correspondents. No communication can be entertained if the name and address of the correspondent is not given, or unless one side of the paper only is written on.]

### "SIR" OR "DOCTOR."

"C. E. H." writes: After reading your article in your journal of August 7th, may I ask your correspondent "Why is a doctor to be called sir?" A nurse should not be addressed as madam. Many women engaged in nursing are ladies by birth and education, and yet they are content with the title that is the correct form of address for a domestic servant, *i.e.*, a woman who takes care of children. It is surely a very trivial matter to make a fuss about, though, so far as I can see there is no reason why a nurse should not in response to remarks address every medical man as "sir," should he wish it. Many doctors, however, are anything but polite to nurses, and they are invariably the gentlemen who complain of lack of courtesy from nurses.

"M. D." writes: I cannot see why a surgeon should be called sir by a nurse any more than a nurse should be called madame by a surgeon. Nurses are not the doctor's servants or employés! They are paid and clothed and fed and housed by the public and the subscribers, not by this "Hospital Surgeon"! It is really time this silly snobbishness (which is making a laughing stock of our profession) should be suppressed, and I trust you will not lend your columns any more to the publication of such letters. For discipline nurses are under the matron or lady superintendent, who is responsible to the house committee only, so beyond carrying out accurately the instructions of the doctors, nurses should in no way consider them to be their superior officers.

"A Late Hospital Sister" writes: I am very glad to read the remarks of "Hospital Surgeon" on the point of etiquette involved in the use of the title "Sir." I notice with regret that this mark of courtesy is declining, not only, I am afraid, among private nurses. Too often the hospital nurse appears to think it beneath her dignity (?). I thoroughly agree with your correspondent that such reluctance is proof of ignorance or bad manners. So far from the use of the little word being derogatory, it is evidence of good breeding, resulting in a sense of the fitness of things. The gentlewoman is not afraid of compromising her position by courtesy and deference to a superior in authority. No other mode of address can take its place. "Doctor" is obviously out of place in addressing a surgeon, and even to a physician is in bad taste, while the constant use of the proper name is not to be tolerated. I trust we may yet see a general return to the more courteous and dignified mode, and to this end I wish that physicians and surgeons would make their opinions felt.

### DISTRICT MIDWIFERY.

"Sister Ada" writes: I cannot allow to pass without some comment the remarks made by "Amy Evans" respecting trained midwives, their unpardonable ignorance, and, as a consequence, the fearful results to the mothers in after life. All I can say is that the term trained is utterly misapplied when speaking of these hopelessly ignorant creatures to whom she refers, for a "trained," educated, and conscientious midwife is an unspeakable blessing to hundreds of poor women, who prefer a woman to a doctor. I have for some years practised as a district midwife. I regard child-bearing as quite a natural event in a woman's life, and have long ago come to the conclusion that the kind of midwife to whom I have referred is the right and proper person to attend the poor in their confinements, and, most assuredly, any woman knowing her work thoroughly is able to foresee any serious complication which might arise at any stage of labour. It is the trained midwife who really knows just the right time when medical aid should be called in and who can successfully and skilfully cope with any emergency which may arise when a doctor is not at hand. "Amy Evans" must surely be referring to the Sairey Gamp class, which, I rejoice to say, is almost a thing of the past. For the sake of thousands of our poor women, may the trained, educated and skilful midwife be ever found in our midst.

### HOME FOR AGED NURSES.

"Policy 2,096" writes: I feel deep sympathy for "Nurse E." and others in her position, but may I suggest to her that it is not too late to avail herself of the Pension Fund? I do not of course know her circumstances, but since she says she is prepared to pay 7s. a week exclusive of board, this implies the possession of a capital which, if applied to the purchase of a paid-up policy in the Fund, would yield enough to keep her from want. At any rate she would get a better return for her money there than by any other means. To go on living on her small capital is fatal. If I knew her address I would write to her, but can only do so now through your paper. I do not think the idea of a central home for nurses one likely to be realised, and I see objections to such a scheme, but an independent income, however small, leaves the owner free to choose her place of abode. In conclusion, I wish to express my personal gratitude to Sir Henry Burdett and the other generous founders of the National Pension Fund.

### THE ROYAL BRITISH NURSES' ASSOCIATION.

Nurse Anne S. Pruett, M.R.B.N.A., writes; I, too, feel that I should like to say a few words to show my loyalty and sympathy to those members of the Royal British Nurses' Association who have been chosen to conduct, guard, and maintain the principles, advantages, and unity of this much maligned association. As Louisa J. Stacy writes, we who have been prompted to join the R.B.N.A. should not withdraw because all is not plain sailing just at present. Our motto is now, "United we stand; divided we fall." Also we should not forget that we have as a stimulus a Royal President, the much-beloved Princess Christian, who is so untiring and practical in her royal patronage of our well-meant society. After a storm, comes a calm. I hope sincerely that we are only staggering after a few shocks, due either to miscarriage of judgment or the pertinacious interference of capricious members, who hinder, instead of aiding, our superiors in council. It behoves us all to keep together, thereby showing what a disciplined, loving corps we are—steadfast and true.

### DISTRICT MIDWIFERY.

Elinor Agnes Bedingfeld writes: I had hoped that an abler pen than mine would have replied to the remarks made by Amy Evans on the above subject, and which I have read with much indignation. With regard to untrained midwives, I am at one with her, but as "A Trained Midwife" who was deemed worthy of being examined four years ago by the Select Committee appointed by the House of Commons to consider the Registration of Midwives Bill, and who has been in successful practice for eighteen years, may I say a few words in answer to her and in defence of "trained midwives"? Firstly, I would remind her that "midwives" have been a recognised necessity since the days of the Patriarchs, and as at the lowest computation 50 per cent. of the mothers of Great Britain are delivered by women of some sort, and will continue to be so for many a long day, I would ask, Is it not better to give them the best article of the kind that can be procured than to leave them to the mercies of women who know absolutely nothing about the matter? Amy Evans despises a "six months' training," but forgets that during that time a midwife is doing nothing but midwifery, whereas a medical student takes midwifery together with all other branches of medicine, and I doubt much if he devotes six months to this subject alone. If A. Evans objects to a trained midwife, what does she say to the young students sent out from the large London hospitals to learn their work on those women who, too poor to pay even a midwife's fee, avail themselves of the free letters granted by the hospitals in order that the students may get practice? To my knowledge, one, having brought the child, prepared to leave, and was surprised when asked by a midwife present if he was not going to finish his work. He had quite forgotten the placenta, and she had to teach him to take it. I personally knew another, then senior house surgeon in a hospital, who had only delivered 30 cases—all normal presentations—and yet he was supposed to be able to undertake midwifery? I met another, sent out alone from a London hospital, who had never seen a breech case, and, did space permit, I could give other instances. How much better were these than a woman who in her training has delivered under proper tuition her dozens of cases? A. Evans forgets, too, that the class of women who train now are very superior to the type of forty years ago, and that to pass the L.O.S. examination one must be fairly educated. Has she ever seen one of the L.O.S. examination papers? Further, there are black sheep in every flock, among doctors as well as midwives, but I protest, writing of the mass of midwives, against the charge she brings that they deliberately give false certificates of still-birth. She insinuated that they knowingly let infants die, and then say they are still-born! A trained midwife would not dare do this for her own reputation's sake, if from no higher motive. Also, when and where has she seen the "grievous results" she writes of from the employment of trained midwives? The late Dr. James Aveling, the trained midwives' friend and advocate, and who, I am proud to say, examined me for my L.O.S. diploma, said, "The art of being a good midwife is to know when to send for a doctor." A trained midwife is only too glad in abnormal cases to shift the responsibility on to the doctor's shoulders when one is procurable; but in lonely country districts, miles, perhaps, from a doctor (I worked where I was four miles in every direction from a medical man), she is obliged often to do her best alone to save life, which would be lost if she waited for the doctor. It is easy to sit in one's room and theorise. But will A. Evans be practical, and say who is to pay the doctor she so kindly wishes to attend in every case? As things are, the trained midwife combines the offices of doctor and nurse, both visiting the patient for ten days, washing and douching her, changing her linen, dressing the baby, &c. And all for a fee of from 5s. upwards. A doctor alone would be 21s., and then the patient must pay a nurse as well. How many of the poor can afford this? A. Evans may think she would prefer an untrained woman. If it came to actual practice I expect she would speedily change her opinion! I can only imagine that she is neither a nurse nor a married woman! To pass on to Query 322, no accurate idea can be formed of the worth of "Obstetric's" work unless she gives the population of her district, whether it is town or country, scattered or compact, and says whether there are other doctors and midwives in the place. Many country doctors would consider forty-five cases per annum a fair number. The only way I know in which to work up a practice is to gain the confidence of the people by your skill and conscientious work, and the committee should aid, by the loan of necessaries and gift of nourishments in necessitous cases.

### TEA-MAKING.

Edward C. Wolferstan writes: Is it not time that a protest was made against the practice of allowing tea to be made by servants? I find it to be almost universal, even at good houses where I take a cup on Sundays on a friendly call, and at others where I stay occasionally. I have drunk tea—made by myself—for many years without apparent injury, and do not believe in the harmful effects so often ascribed to it. But then it must not be left to servants unless they are properly instructed as to quality; and I find that practically the good old rule, "One teaspoonful for each drinker and one for the pot," works out admirable results. However, let me state my late experience. On Sunday, July 11th, I paid a visit to friends in St. John's Wood, but, arriving rather late, the servant was ordered to make fresh tea for me, which was done. I did remark that the flavour was rather strong, but, thinking nothing of this at the time, drank my usual two or three cups, nor did I feel any immediate ill-effects therefrom, except that three hours afterwards I found myself quite unable to eat any dinner. But for two days thereafter I found myself attacked by the most intense prostration and weakness, followed by diarrhœa, which, in spite of medical advice and the greatest care in diet, lasted me a fortnight. What, I have no doubt, happened is this: The servant, from mere laziness, merely added fresh tea—and perhaps several spoonfuls—to the already strong dregs in the pot, thus simply poisoning me with a decoction which, when of such strength, is, I am certain, as deadly as any drug in the Pharmacopœia. I could quote several other instances personal to myself of servant-made tea, but forbear to encroach further on your space.

### The Book World for Women and Nurses.

[We invite Correspondence, Criticism, Enquiries, and Notes on Books likely to interest Women and Nurses. Address, Editor, The Hospital (Nurses' Book World), 28 & 29, Southampton Street, Strand, London, W.C.]

Notes on Medical Nursing. By the late James Anderson, M.D., F.R.C.P., Assistant Physician to the London Hospital, Physician to the National Hospital for the Paralysed and Epileptic, Lecturer on Pathology at the London Hospital, &c., &c. Edited by Ethel F. Lamport, Associate of the Sanitary Institute and of the British Institute of Public Health; formerly Sister at the London Hospital. Third edition. Crown 8vo, pp. 184. Price 2s. 6d. (H. K. Lewis, 136, Gower Street, W.C.)

That this little book has reached its third edition in one and a half years is the most convincing testimony that can be produced of its merit and increasing popularity. There is no other work we know of, that goes so thoroughly into the scientific aspects of nursing, while at the same time care is taken not to loose sight of the practical details so essential to success. Miss Lamport, to whose skill and erudition we are indebted for the useful form in which these valuable lectures now appear, is to be congratulated on the success which has attended her labour of love. A careful perusal of its pages will amply repay the student, and there are suggestions made which it would be well if they could be carried into effect. For instance, in Lecture II., among some excellent hints on feeding the patient, we find the following advice: "Do not leave your patient alone during his meals; a nurse with tact can always find some little thing to do about the room so as not to actually watch her patient, and few things are more distressing than to have someone sitting and watching you all the time you are eating." This is too true, and yet how few nurses think of it. The same principle applies to special cases on night duty, where a nurse should always busy herself apparently with some needlework or a book so as to obviate the disagreeable sensation that would naturally result from a person sitting idly by the bedside. Yet another extract, for it is worthy of all the emphasis we can give it. "Do not try to cram a dying patient with food, as it worries him and renders his last moments more painful than they would otherwise be. A dying man will swallow any liquid that is given him, but he cannot digest it, as thus it merely remains in the stomach in an undigested form, and causes discomfort. Therefore do not try to feed a dying person." The chapters on temperature, infection, and fevers contain much useful information, especially with reference to the complications that a patient is liable to, after the acute stage of the disease has passed away. For instance, after scarlet fever there is the tendency to inflammation of the kidneys, not necessarily the results of cold, but as often due to the extra work thrown on these organs in ridding the system of the poison of the disease. It is recommended, therefore, that very little nitrogenous food be given till after the third week, so as to give the kidneys as much rest as possible. The description of the various rashes met with in infectious diseases is extremely valuable, as likewise are the recommendations laid down for cleansing and disinfecting the room, &c. Not the least useful part of the book is a short glossary of technical terms found at the commencement.

### For Reading to the Sick.

### "SUBMISSION."

If so be that we suffer with Him, that we may be also glorified together.—*Romans* viii. 17.

**Verses.**

When clouds awoke by Sorrow's wand
Come o'er the soul in heaviness,
Sweet is the thought of Heaven beyond,
A cave of holy quietness,
Like clay beneath the waters seen,
Housed in a deep and blue serene,
A strange unearthly deep repose,
'Mid hanging rocks, all calmly laid,
But touched not by their darkening shade,
The Tower of Heaven beyond earth's woes.
—*Isaac Williams.*

In the cruel fire of sorrow
Cast thy heart, do not faint or wail!
Let thy heart be firm and steady,
Do not let thy spirit quail!
But wait till the trial is over,
And take thy heart again;
For as gold is tried by fire,
So a heart must be tried by pain.—*A. Procter.*

God will never leave thee,
All thy wants He knows,
Feels the pains that grieve thee,
Sees thy cares and woes.
Raise thine eyes to heaven
When thy spirits quail,
When by tempests driven
Heart and courage fail.—*Frances E. Cox.*

Though by sorrows overtaken,
Lord, Thy servants seem forsaken,
Thy Almighty Hand we know
Blendeth love with human woe.
Love, unlike all worldly pleasures,
Wraps in grief its golden treasures;
And to meek and wounded hearts
Deep and holy love imparts. —*A. Herbert.*

**Reading.**

They who have truly learned patience have been learning submission at the same time. But there is a difference between patience and submission. Patience is lying in a still posture before God, waiting His commands and His time; patience is the refraining from all efforts and vehemence and eagerness—everything that would go between, or outrun, the leadings of God. But submission is the actual surrender of ourselves, our bodies, souls and spirits, our wills, into the hands of God; the giving up all; the practical saying of the heart and actions, that He knows better than we do, that He chooses better for us, that we yield all to Him, and are pleased with what pleases Him. Submission is of very slow growth; it wholly opposes and resists nature. We have strong wills of our own, and they strive fiercely with the will of God. Even our very prayers are the expressions of self-will; if we do not dare to say the words, yet do they not often mean *my* will be done? . . . The greatest help to submission is to receive everything straight from God. Do not look at second causes, never suffer yourself to do that; do not look back to the beginning of your illness and think how it first came on; how it might, perhaps, have been avoided; how it might have been removed in its early stage; how circumstances have aggravated it, and do so still. You have nothing to do with these things. God sent you your illness at first. God placed you where you now are, and exactly in the very circumstances that you find yourself. Bear this always in mind.—*From Manual of Sickness: Its Trials and Blessings.*

### Notes and Queries.

*How to Become a Nurse.*

(344) Would you kindly inform me how to become a nurse? I was 21 last April.—*M. Roberts.*

Read Honnor Morten's "How to Become a Nurse" (Scientific Press, 28 & 29, Southampton Street, Strand), price 2s. 6d. You must begin work either at a children's hospital or an infirmary, on account of your age. Chapter IV. of the book recommended gives an alphabetical list of chief training schools in London and the provinces, with the age at which probationers are received.

*A List of Cottage Hospitals.*

(345) Will you kindly tell me if there is any inexpensive book published besides Honnor Morten's "How to Become a Nurse" which gives a list of the cottage hospitals?—*Perry.*

"Burdett's Hospitals and Charities," price (net) 3s. 9d., from Scientific Press, 28 & 29, Southampton Street, Strand.

*"A Book on Nursing."*

(346) I would like to get a book on nursing that goes into the causes as well as the effects and treatment of diseases. I should be obliged if you can tell me of a medical dictionary giving more information than Hoblyn, but simpler than Quain.—*Crouch End.*

Our rule is not to answer queries unless accompanied by name and address of sender, as a pledge of good faith.

*The Guardians' Power.*

(347) Would you kindly tell me if the Guardians have power to call upon a probationer nurse to resign after having served nearly two years without once being reported for neglect of duty or disloyalty to the superior officer, simply from malicious tittle tattle by a nurse in disgrace herself. (2) Why should a nurse's career be spoiled?—*Nurse A. Arthey.*

The Guardians have the power to ask a nurse to resign. (2) A careful and obedient nurse is not likely to have her career ruined by one act of injustice.

*A Medical Dictionary.*

(348) Will you kindly tell me of a larger dictionary than that by "Honnor Morten"?—*Sister Gertrude.*

"Hoblyn's Dictionary of Medical Terms," published by Whittaker and Co., Paternoster Square, price 10s. 6d., is a very useful book of the kind.

*Nurses at the Riviera.*

(349) I have a nurse who would like to go to the Riviera to nurse this winter. Would you be so good as to advise me how and to whom to apply?—*Matron.*

Apply for particulars to the Directress, Hollond Institution, 1, Tavistock Chambers, Bloomsbury, W.C. The headquarters of this institution are at Villa Berthe, Rue d'Italie, Nice. Miss Bryant has a Nurses' Institute at 19, Via Vittorio Emanuele, San Remo; and Mrs. Dan Norris takes nurses every winter to her Invalids' Home, Villa d'Ormesson, Cannes. It is important that suitable work should be obtained for the nurse before she leaves England, and also that she has some guarantee against dismissal at a week's notice.

*Nursing in America.*

(350) What are a nurse's prospects in America? Does she earn a better salary by private work than in England?—*A Nurse.*

Private nurses are provided in America by means of directories (see pp. 95, "How to Become a Nurse," by Honnor Morten, Scientific Press, 28 and 29, Southampton Street, Strand). English nurses have many difficulties to encounter, and miss home comforts. The pay is from £3—£4 per week. A nurse must either go out to work or be possessed of sufficient capital to keep her until she obtains it, as to undertake any other kind of work is considered derogatory. Great care is needed in making any move in this direction.

*Nursing Prospects in Bermuda.*

(351) I would be much obliged if you will tell me if you think there would be work in Bermuda for a thoroughly qualified nurse and midwife. Two of my friends are going in October. Do you think there would be work for me as a third?—*Nurse Madge.*

Mrs. Francis T. Piggott, The Colonial Nursing Association, Imperial Institute, S.W., would probably be able to inform you as to the demand for nurses in Bermuda, the cost of living, and the prospects of a cottage hospital. You should also write to the Agent-General for the Colony, Emigrants' Information Office, 31, Broadway, Westminster.

*How to Become a Probationer.*

(352) I am anxious to become a probationer at a cottage or small hospital. (2) What are the duties of a probationer? (3) Would it be possible to get a small salary for the first year? (4) Is there any correspondence class with which I could work?—*Pro.*

See Honnor Morten's book, "How to Become a Nurse," price 2s. 6d., the Scientific Press, 28 & 29, Southampton Street, Strand, which answers all these questions fully.

*The Baths at Droitwich.*

(353) I should be glad if you could inform me what would be the cost for board, lodging, and brine baths at Droitwich or elsewhere. I am troubled with rheumatism, and think that the baths would relieve me.—*District Nurse.*

Good board and a very nice lodging can be obtained at Mrs. Baces', Elm Villa, Droitwich, for 25s. a week. A bed-room with use of sitting-room can be had at Mrs. Partridge's, White Rose Cottage, for 10s. a week.—*L. H.*

